# Genotypic variants of the tetrahydrobiopterin (BH4) biosynthesis genes in patients with hyperphenylalaninemia from different regions of Iran

**DOI:** 10.1002/mgg3.2294

**Published:** 2023-10-11

**Authors:** Seyed Reza Kazemi Nezhad, Pegah Namdar Aligoodarzi, Golale Rostami, Gholamreza Shariati, Hamid Galehdari, Alihossein Saberi, Alireza Sedaghat, Mohammad Hamid

**Affiliations:** ^1^ Department of Biology, Faculty of Science Shahid Chamran University of Ahvaz Ahvaz Iran; ^2^ Department of Molecular Medicine, Biotechnology Research Center Pasteur Institute of Iran Tehran Iran; ^3^ Department of Medical Genetics, Faculty of Medicine Ahvaz Jundishapur University of Medical Sciences Ahvaz Iran; ^4^ Department of Genetics, Faculty of Sciences Shahid Chamran University of Ahvaz Ahvaz Iran; ^5^ Department of Endocrinology Ahvaz Jundishapur University of Medical Sciences Ahvaz Iran

**Keywords:** metabolism disorder, non‐*PAH* deficiency, Sanger sequencing, tetrahydrobiopterin (BH4)

## Abstract

**Background:**

Hyperphenylalaninemia (HPA) is a metabolic disorder classified into phenylalanine‐4‐hydroxylase (*PAH*) and non‐*PAH* deficiency. The latter is produced by mutations in genes involved in the tetrahydrobiopterin (BH4) biosynthesis pathway and *DNAJC12* pathogenetic variants. The BH4 metabolism, including de novo biosynthesis involved genes (i.e., guanosine 5′‐triphosphate cyclohydrolase I (GTPCH/*GCH1*), sepiapterin reductase (SR/*SPR*), 6‐pyruvoyl‐tetrahydropterin synthase (PTPS/*PTS*)), and two genes that play roles in cofactor regeneration pathway (i.e., dihydropteridine reductase (DHPR/*QDPR*) and pterin‐4α‐carbinolamine dehydratase (PCD/*PCBD1*)). The subsequent systemic hyperphenylalaninemia and monoamine neurotransmitter deficiency lead to neurological consequences. The high rate of consanguineous marriages in Iran substantially increases the incidence of BH4 deficiency.

**Methods:**

We utilized the Sanger sequencing technique in this study to investigate 14 Iranian patients with non‐*PAH* deficiency. All affected subjects in this study had HPA and no mutation was detected in their *PAH* gene.

**Results:**

We successfully identified six mutant alleles in BH4‐deficiency‐associated genes, including three novel mutations: one in *QDPR*, one in *PTS*, and one in the *PCBD1* gene, thus giving a definite diagnosis to these patients.

**Conclusion:**

In this light, appropriate patient management may follow. The clinical effect of reported variants is essential for genetic counseling and prenatal diagnosis in the patients' families and significant for the improvement of precision medicine.

## Graphical abstract

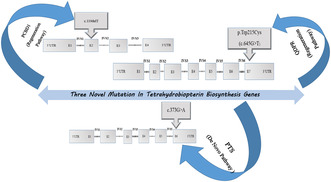

## 1 INTRODUCTION

Phenylketonuria (PKU, # 261600) is the most common form of HPA. It is a hereditary autosomal recessive metabolic disorder of newborns belonging to the aminoacidopathies, which is most often caused by missense mutations in the hepatic *PAH* gene (Su et al., 2019). This abnormality can alter cerebral myelin, serotonin, noradrenaline, and dopamine levels in the brain. The disease's clinical hallmarks include intellectual disability, microcephaly, delayed speech, seizures, behavioral abnormalities with varying severity, seizures, autism, eczema, rash, and psychiatric symptoms (Blau et al., 2010). Well‐timed detection of PKU is required to conduct the correct therapy to avoid these complications. The lack of diagnosis and treatment during the first year of life and the resultant neurotoxic effects of excess phenylalanine (Phe) can cause the reduction of a child's intelligence quotient (IQ) capacity by 50% and complicate future treatments (Burton et al., 2013). The frequency and distribution of HPA vary among different nationalities and ethnic groups worldwide. For example, the HPA rate for Chinese is 1 per 15,415 births and for Japanese is 1 per 143,000 births. The prevalence of HPA is about 1:2600 in Turkey (Su et al., 2019). Also, the HPA prevalence ratio varies among the Iranian ethnic groups (Heidari et al., 2021). A systematic review and meta‐analysis study indicated that HPA prevalence was estimated to be 38/100,000 and 43.3/100,000 in female and male infants, respectively (Shokri et al., 2020). According to the screening program findings, 2%–3% of mentally disabled patients kept in sanitariums suffer from PKU (El‐metwally et al., 2018; Habib & Hossein, 2010; Vallian et al., 2003). The conventional treatment for PKU involves diet therapy aimed at limiting the consumption of Phe. On the other hand, the classical treatment for HPA relies on monitoring Phe levels, and in general, no specific diet treatment is required. However, several alternative methods have appeared, such as sapropterin therapy, pegvaliase enzyme replacement therapy (Ashe et al., 2019; Blau et al., 2022; Hydery & Coppenrath, 2019), and using probiotic bacteria modified to process Phe in the patients' intestines (Isabella et al., 2018). HPA is also caused by defects in the genes that encode the proteins involved in BH4 biosynthesis and metabolism. BH4 (6R‐L‐erythro‐5,6,7,8‐tetrahydrobiopterin) is the essential cofactor of Phe, tyrosine, the two isoforms of tryptophan hydroxylase (TPH 1/2), alkylglycerol monooxygenase (AGMO), and the three isoforms of nitric oxide synthase (NOS 1–3). BH4 deficiency leads to increased blood Phe levels and cognitive and motor disturbances owing to reduced syntheses of dopamine and serotonin (Werner et al., 2011). In response to increased Phe concentration in the blood, the structure of PAH changes to its activated form stabilized by BH4. As a large neutral amino acid (LNAA), Phe competes with other LNAAs for transporters in the blood–brain barrier (BBB). The increased blood Phe concentration or insufficient levels of non‐Phe LNAAs in the central nervous system can reduce the synthesis of proteins essential for proper brain development and function, thus leading to severe neurological disorders (Hofman et al., 2018). Treatment with BH4 resulted in significant (at least 30%) and sustained reductions in Phe blood concentrations and increased dietary Phe tolerance in responsive PKU patients. However, besides *PAH* deficiency, some HPA cases are caused by impaired BH4 pathways. Autosomal recessive mutations cause these rare variants of BH4 biosynthesis genes, including DHPR (OMIM: 261630), GTPCH (OMIM: 233910), PTPS (OMIM: 261640), and PCD (OMIM: 264070) (de Souza et al., 2018). According to the pediatric neurotransmitter disorders database PNDdb, about 324 variants have been found in the *GCH1* gene, of which 11 are autosomal recessive with hyperphenylanemia and neurotransmitter deficiency, 295 are dominant, and the phenotype of 18 variants remains unclear. Autosomal recessive variants found in the *SPR* gene (104 variants, OMIM: 612716) present without hyperphenylalaninemia, while variants seen in the *PTS* (199 variants), *PCBD1* (32 variants), and *QDPR* (141 variants) genes lead to HPA along with central monoamine neurotransmitter deficiency (Himmelreich et al., 2021).

Differentiating *PAH* deficiency from the ВН4 deficiencies (BH4Ds) is crucial because the Phe‐restricted diet is not a suitable therapy for patients with rare HPA forms, and these patients often receive the Phe‐restricted diet as the only treatment without differential diagnostics. The measurement of blood Phe (plasma/serum) is a crucial HPA diagnostic test, but it cannot differentiate an HPAВН4 from a *PAH* deficiency (Blau et al., 2010). In contrast to PKU, BH4 deficiencies are complicated by the depletion of the brain neurotransmitters dopamine, serotonin, and norepinephrine, which can be overcome by oral supplementation with the neurotransmitter precursors L‐Dopa and 5‐hydroxytryptophan (5 HT) (Opladen et al., 2012). This study aimed to analyze Iranian patients with rare forms of HPA and confirm the definitive diagnosis of their disease to begin appropriate treatment.

## 2 MATERIALS AND METHODS

### 2.1 Ethical compliance

Informed consent was obtained from the guardians of all patients to participate in the study. The study was approved by the Pasteur Institute of Iran Ethics Committee [Ethical approval no. IR.PII.REC.1397.65].

### 2.2 Patients

DNA samples of 14 unrelated probands with a “hyperphenylalaninemia” diagnosis referred to the Kowsar Genetic Laboratory and Pasteur Institute of Tehran, Iran, and the Narges Genetic Laboratory of Ahwaz, Iran, were included in this study. The probands were selected based on their clinical, biochemical, and genetic screening results for HPA. According to the HPA patient's genotyping program, all the probands were analyzed for *PAH* gene variants, and no variants were detected in this gene. Most of these patients were recognized during the newborn screening program (NBS).

### 2.3 DNA amplification and sequencing

Salting‐out was used for extracting patients' DNA samples from their blood. PCR experiments were performed with 100 ng of genomic DNA, 15 pmol of each specific primer pair, and 0.2 μL of AmpliTaq DNA polymerase (Applied Biosystems, Austin, TX, USA). PCR products were purified with the QIAquick PCR Purification Kit and sequenced in both directions using BigDye terminator v1.1 chemistry on an ABI PRISM 3130xl Genetic Analyzer (Applied Biosystems, Austin, TX, USA). DNA sequencing was performed on the DNA coding sequence, and exon boundaries, and partially covered 5′ and 3′ untranslated regions of the *PTS*, *PCBD*, and *QDPR* genes. These regions were amplified in 15 amplicons (Tables 1, 2, 3). Primers were designed using the Primer 3 program (http://frodo.wi.mit.edu/). A total of fifteen PCR primers were designed based on the ensemble reference sequence and transcript ID of *PTS* (NM_000317.2, ENST00000280362.8), *PCBD* (NM_000281.3, ENST00000299299.4), and *QDPR* (NM_000320.2, ENST00000281243.10) genes. All primers selected had a melting temperature between 58 and 60.5°C, (using the Biomaths Calculator program: http://www.promega.com/techserv/tools/biomath/calc11.htm), a purine:pyrimidine content close to 1:1, and their lengths ranged between 18 and 23 nt. The primer sequences were checked to avoid similarities with other loci or repetitive sequences in the genome by using the BLAST program to confirm their specificity for the respective sequences (http://blast.ncbi.nlm.nih.gov/Blast.cgi). Secondary structures, hairpin structures, ΔΔG of self‐ and hetero‐dimers, and potential primer‐primer interactions were predicted using the OligoAnalyzer 3.1 tool at http://eu.idtdna.com/analyzer/Applications/OligoAnalyzer/ and Gene Runner Version 6.3.03. Additionally, each designed primer was validated by the SNP checker (https://genetools.org/SNPCheck/snpcheck.htm) to investigate the possible presence of single nucleotide polymorphisms (SNPs) in their sequence. DNA alignment with the reference sequence and variant analysis were carried out using Chromas software.

**TABLE 1:** List of *QDPR* exon amplification primers and product sizes (bp).

*QDPR* primer						
Exon number	Primer sequences		Length	GC content	TM	Product size
1	F	CGAAGTTACAGTCCCTCCGG	20	60	59	383
	R	CACCTTCCTCTAGACTGCCC	20	60	59	
2	F	GGCCAACTCCCCTCATTTCT	20	55	60	247
	R	AACATACAGCCAGTGGTCAC	20	50	58	
3	F	TCCGTAAAGATGCTAGCCTGT	21	47.5	59	230
	R	CCCAATCCTTGTCAGCTGGA	20	55	60	
4	F	GCTGTAGATAGGGACCTGGG	20	60	58.5	300
	R	GCAACCCCACTCCACAGATA	20	55	59	
5	F	TTCCCATCCCCTCTTTCTCG	20	55	59	393
	R	GTCGCCTGTGGAAAGCTAC	19	57.8	59	
6	F	GTCCTGCTGTATGTTTGCGT	20	50	59	324
	R	ATGCATCCTCAGAGTCCCAG	20	55	59	
7	F	ACAAGCCGTCACTGTGAAG	19	52.6	58	442
	R	TATGCAGAGCCCTCCCTATG	20	55	58	

**TABLE 2:** List of *PCBD*1 exon amplification primers and product sizes (bp).

*PCBD*1 primer						
Exon number	Primer sequences		Length	GC content	TM	Product size
2	F	ATTGCTCAAAGACCTGCTGC	20	50	59	336
	R	ATGCTTTGTAAGGTGACCCC	20	50	58	
3	F	CCTGCGTCTAGGCCTTGAAT	20	55	60	194
	R	GAGCACAACAGAGGCACAC	19	58	59	
4	F	CAGCAGCTAGTGACTCCCTC	20	60	60	619
	R	AGAGCCTGAGACCAAGTGAT	20	50	59	

**TABLE 3:** List of *PTS* exon amplification primers and product sizes (bp).

*PTS* primer						
Exon number	Primer sequences		Length	GC content	TM	PCR product
Exon 1	F	GAGACGCACTTCCTAGGGG	19	63	59	352
	R	GTCCGAAGCCCCACGAAG	18	66	60.5	
Exon 2	F	GGGGTTTGAATGTGATACTTGTG	23	43	58	229
	R	GTGTCCGTAAGTTTTCCCATTCT	23	43	59	
Exon 3	F	TGTGTCTTGCTTTGGTTGCT	20	45	58.5	308
	R	AACAGTTTTCAACACAGAATCCA	23	35	57	
Exon 4	F	AGTCTCTGCACATTGTACTGC	21	47	58	180
	R	AGCACATAATAAGCACCCAACA	22	40	58	
Exon 5,6	F	CGTAGGTGTGACAGTGTTATGG	22	50	59	784
	R	TTTGTGCAATGCTAACCCCA	20	45	58	

### 2.4 Data interpretation

Databases examined for detected variants included the locus‐specific database PNDdb (http://www.biopku.org/home/pnddb.asp), ClinVar (https://www.ncbi.nlm.nih.gov/clinvar/), Ensemble (https://ftp.ensembl.org/pub/release‐108/variation/gvf/homo_sapiens/), and LOVD (https://databases.lovd.nl/shared/genes). If variants were not recorded in these databases, they were also searched in population databases such as Iranom, dbVar, dbSNP, and gnomAD to determine their frequency and registration status. And finally, if a variant was not in these databases, it was considered a new variant. On the other hand, as an identified variant was described in more than two literary sources as a cause of the disorder, it was classified as pathogenic. Therefore, the putative pathogenicity of the nonsynonymous gene variants of *PTS*, *PCBD*, and *QDPR* genes was inspected by querying the dbNSFP version 3.0b2a database for functional prediction and annotation of all potential nonsynonymous single‐nucleotide variants (nsSNVs) in the human genome. In particular, SIFT, PROVEAN 1.1, PolyPhen‐2 v2.2.2, MutationTaster2, and FATHMM v2.3 predictions were extracted for each single missense variant. When the patients' mutation loci were detected, they were confirmed by PCR and bidirectional Sanger sequencing on the parental sample.

## 3 RESULTS

### 3.1 BH4 deficiency *QDPR* gene

Four probands out of 14 had 4 variants in the QDPR gene, out of which only 3 were previously described in literature sources: p.Leu132=(c.396G>A), p.Tyr150Cys (c.449A>G), and p.Arg221* (c.661C>T), and 1 variant was not reported: p.Trp215Cys (c.645G>T). Except for the p.Arg221* variant in the *QDPR* gene, a nonsense variant, all variants identified in this gene were missense. Moreover, in terms of the distribution of variants, all variants were located in the exonic regions. Of all the variants detected in the *QDPR* gene, two variants in exon 7, one variant in exon 5, and one variant in exon 4 were located.

The variants were classified as pathogenic (P) or likely pathogenic (LP) based on the pathogenicity criteria (Table 4). Both parents in the four families with detected *QDPR* variants were heterozygous, confirming the variants' transposition (Table 5). This novel variant was confirmed in both parents' *QDPR* sequencing results. Thus, it was confirmed that the fourth proband is a non‐classic PKU patient. This variant was in a homozygous state in proband. The Trp 215 Cys (W215C) variant was detected in a heterozygous state during the family analysis.

**TABLE 4:** *QDPR*, *PTS*, *PCBD1* variants.

Position in cDNA	Position in protein	Prevalence %	Pathogenicity criteria^a^	Variant description	Variant pathogenicity	
					PolyPhen	PROVEAN
*QDPR* variants (NM_000320.2) 576G>A	Leu132 Leu	7.1	–	Synonymous	–	–
485A>G	Tyr150 Cys	7.1	LP/PP	Missense	Likely pathogenic	Deleterious
825G>T	Trp 215 Cys	7.1	PP	Missense	Likely pathogenic	–
841C>T	Arg221*	7.1	–	Nonsense	–	–
*PTS* variants (NM_000317.2)						
452G>A	Gly125Arg	7.1	BP	Missense	Benign	Deleterious
*PCBD1* variants (NM_000281.3)						
370delT	Phe40Serfs*11	7.1	PVS	Frame shift	Probably damaging	Deleterious

^a^ Pathogenicity criteria abbreviations (PVS, PS, PM, PP) are given by the data interpretation recommendations (PVS, pathogenic very strong; PS, pathogenic strong; PP, pathogenic supporting; PM, pathogenic moderate); Bp, benign supporting; LP, likely pathogenic variant (Richards et al., 2015).

**TABLE 5:** Genotypes of patients with detected variants in the *QDPR*, *PTS* and *PCBD1* gene, family examination results.

Allele1	Allele2	Exon/intron	Method	Family examination
*QDPR* gene				
c.396G>A	c.396G>A	Exon 4	*QDPR* sequencing	Carriage confirmed in the mother and the father
c.449A>G	c.449A>G	Exon 5	*QDPR* sequencing	Carriage confirmed in the mother and the father
c.645G>T	c.645G>T	Exon 7	*QDPR* sequencing	Carriage confirmed in the mother and the father
c.661C>T	c.661C>T	Exon 7	*QDPR* sequencing	Carriage confirmed in the mother and the father
*PTS* gene				
c.373G>A	c.373G>A	Exon 6	*PTS* sequencing	Carriage confirmed in the mother and the father
*PCBD1* gene				
c.119delT	c.119delT	Exon 2	*PCBD1* sequencing	Carriage confirmed in the mother and the father

### 3.2 BH4 deficiency *PTS* gene

One proband out of 14 had one variant in the *PTS* gene that was novel and not previously described: c.373G>A, g.712G>A, and Gga>Aga. In connection with the *PTS* gene, the identified variant was located in exon 6 and was a missense variant.

The pathogenicity prediction criteria were classified as “deleterious” and “benign” according to the PROVEAN and PolyPhen software, respectively. This variant (p. Gly125Arg) could be attributed to the PKUBH4C phenotype. The detected variants' pathogenicity specifications are presented in Table 4. Moreover, the family analysis carried out for the family with HPABH4C showed that both parents were heterozygous carriers (Table 5).

### 3.3 BH4 deficiency *PCBD1* gene

Sequencing found a deletion in exon 2 of the *PCBD*1 gene. One proband out of 14 had one variant in the *PCBD1* gene, which was novel and not previously described: c.119delT, Phe40Serfs*11. Because this mutation caused a frameshift in the protein codon reading, a termination codon appeared after changing 10 amino acids. Frameshift variant c.119delT leads to the replacement of phenylalanine with serine at position 40 and premature termination of translation at 10 amino acids after the variant site (position 50), which results in a truncated PCD protein.

The detected variants' pathogenicity specifications are presented in Table 4. Family analysis was carried out for the family with HPABH4D, and both parents were confirmed to be heterozygous carriers (Table 5). In this study, comprehensive clinical information is not readily available for patients with HPA, and pre‐treatment pterin levels are not known for all patients, so the clinical information for patients with HPA is incomplete but presented in Table 6.

**TABLE 6:** Laboratory data for patients.

Patiant	*PAH* diagnosis	Phe (B) level(μmol/L)	Bio (U) (mmol/mol creatinine)	Neo (U) (mmol/mol creatinine)	Molecular diagnosis
P1	Normal	282	10.13	1.61	*QDPR*, p.Leu132=
P2	Normal	540	6.26	1.18	*QDPR*, p.Tyr150 Cys
P3	Normal	357	1.2	1.12	*QDPR*, p.Trp 215 Cys
P4	Normal	600	6.7	4.8	*QDPR*, p.Arg221*
P5	Normal	430	0.07	24.08	*PTS*, p.Gly125Arg
P6	Normal	1325	nd	1.6	*PCBD1*, p.Phe40Serfs*11
P7	Normal	827	–	–	*PCBD*, *QDPR*, and *PTS* genes are normal
P8	Normal	900	0.25	0.23	*PCBD*, *QDPR*, and *PTS* genes are normal
P9	Normal	621	–	–	*PCBD*, *QDPR*, and *PTS* genes are normal
P10	Normal	1200	–	–	*PCBD*, *QDPR*, and *PTS* genes are normal
P11	Normal	841	10.59	4.86	*PCBD*, *QDPR*, and *PTS* genes are normal
P12	Carier for c.907T>G SNP	480	–	–	*PCBD*, *QDPR*, and *PTS* genes are normal
P13	Normal	868	–	–	*PCBD*, *QDPR*, and *PTS* genes are normal
P14	Normal	510	10.84	11.04	*PCBD*, *QDPR*, and *PTS* genes are normal

*Note*: Phe (B) phenylalanine in blood, Neo (U) neopterin in urine, Bio (U) biopterin in urine, nd not detected.

## 4 DISCUSSION

After thalassemia, PKU (OMIM#261600) is considered the most common autosomal recessive disease in the Iranian population, with consanguinity among carrier couples being the leading risk factor (Moghadam et al., 2018). A mandatory newborn screening program (NSP) for PKU was launched in Iran in 2006, and its purpose was to reduce mental retardation, physical disability, and medical and nonmedical costs (Heidari et al., 2021). BH4 deficiency is classified among the non‐PKU hyperphenylalanineamia group of pediatric neurotransmitter disorders, the diagnostic and treatment measures of which are different from those of *PAH* deficiency. Therefore, a differential diagnosis is critical to distinguish infants with *PAH* deficiency from those with HPA‐BH4 deficiency (Fernández‐Lainez et al., 2018). BH4 deficiency could be detected through the NSP between 2 and 14 days after birth, and the diagnostic test is becoming available in most developed countries (Opladen et al., 2012). However, this differential diagnostic test is not routinely performed in Iran, contradicting the purpose of the NSP (Heidari et al., 2021). HPA‐BH4 deficiency is the cause of approximately 30% of all cases of HPA in Taiwan (Niu, 2011). By contrast, the estimated incidence of BH4 deficiency in Caucasians is 1%–2% of all patients with HPA. In France, BH4 deficiency causes 2.8% of HPA incidence cases (Dhondt, 2010), while it causes 10.1% of the HPA cases in Shandong province, China. Thus, the incidence of BH4 deficiency in various populations is subject to ethnic and geographic variations (Han et al., 2015).

The ВН4‐deficient HPA is classified into four types: the variants causing HPAВН4A are located on the *PTS* gene; those causing HPAВН4B are on the GCH1 gene; the variants responsible for HPAВН4C are on the *QDPR* gene; and HPAВН4D arises from the *PCBD*1 gene variants (Dudešek et al., 2001). The most frequent of them, HPABH4A (approx. 54% of all HPABH4) and HPABH4C (approx. 33% of all HPABH4) are caused by the *PTS* and *QDPR* gene variants, respectively, which are characterized by a deficit in central monoamine neurotransmitters (Han et al., 2015).

The *PTS* gene contains six exons and has been mapped on chromosome 11 (11q23.1) (Himmelreich et al., 2021). The PTPS functional enzyme is a hexamer of identical subunits. The three histidine residues 23, 48, and 50 formed a transition metal binding site in each subunit and bound with Zn(II). This form is responsible for the enzymatic activity (Bürgisser et al., 1995). Site‐directed mutagenesis of each of these three histidine residues results in a complete loss of metal binding and enzymatic activity.

In 1994, Thony et al. characterized three variants of PTPS deficiency (Thöny et al., 1994). Currently, 141 variants are listed in the database of gene variants causing BH4 deficiencies, distributed across all six exons and five introns. Global HPABH4 prevalence varies greatly. In 2018, Li et al. reported four novel mutations in the *PTS* gene among 44 BH4‐deficient Chinese patients (Li et al., 2018). In 2021, Gundorova et al. showed that the incidence ratios of HPABH4A and HPABH4C were 83.3% and 13.3%, respectively, among the studied Russian patients (Gundorova et al., 2021). In this study, we have shown the novel p.Gly125Arg variant located in exon 6. This variant resulted in the conversion of glycine to arginine at position 125 of the PTPS protein. One non‐polar amino acid is replaced by a polar amino acid with a positive charge; glycine is smaller than arginine (the smallest amino acid); thus, an alteration in the size of an amino acid in a sensitive position can affect the interactions that naturally occur between that position and other monomers or other parts of the same protein. On the other hand, according to the UniProt database, this protein has three phosphorylation sites: positions 19 and 28, where serine amino acids are located, and position 128, where tyrosine amino acid is located. Position 128 is close to the position where the mutation occurred. As a result of this mutation, protein phosphorylation may be affected.

The in silico pathogenic predictions for p.Gly125Arg were inconsistent: benign (PolyPhen), disease‐causing (Mutation Taster), and deleterious (PROVEAN), while, according to the American College of Medical Genetics and GenomicsAssociation for Molecular Pathology (ACMG‐AMP) recommendations, it was considered likely pathogenic (>90%) and led to the development of BН4‐deficient hyperphenylalaninemia A.

This mutation is not registered in the dbSNP, ClinVar, ExAC browser, Ensembl, and 1000 Genomes Project databases. However, it is registered in the Iranome (http://www.iranome.com/) and PNDdb (http://www.biopku.org/home/pnddb.asp) databases. The Iranome database shows that the allelic frequency of this mutation is 0.006274. It is interesting to note that this mutation has been observed in a heterozygous individual from the Iranian Arab race in the Iranome database. Additionally, the patient examined in our studies, who also has this mutation, is from the Arab race of southern Iran and is homozygous due to consanguineous marriage. This mutation has not been observed in other Iranian races such as Azeri, Baloch, Kurd, Lur, Persian, and Turkmen. This may indicate that this mutation is spreading within this particular breed.

Moreover, a study conducted by Khamooshian et al. in connection with *PTS* gene variants in Iran reported this mutation (Khamooshian et al., 2022). This study showed that 59.2% (32 of 54) of the mutations found in the *PTS* gene in the Iranian race were found in the intronic region. This rate is higher than the 19.59% mutation rate of the intron region that was taken from the PNDdb database (39 out of 199). Therefore, when examining mutations in this gene, attention should be paid to the intronic regions as well. In Khamooshian et al.'s study, it was shown that this mutation has a deleterious effect on seven or more tools for predicting the effect of mutations (Khamooshian et al., 2022).

The enzymatic recycling of the BH4 cofactor heavily relies on the vital role of the DHPR enzyme. In this process, the DHPR utilizes NADH to provide two hydrogen atoms to 7,8‐dihydrobiopterin (BH2), allowing for the recovery of BH4 (Breuer et al., 2019). Compared to other BH4 deficiencies, DHPR‐deficient patients show a higher frequency of severe neurological symptoms, including hypotonia, movement disorders (mainly dystonia), microcephaly, epilepsy, the brain atrophy caused by the BH2 accumulation, and the inhibition of nitric oxide synthase and aromatic acid hydroxylases due to DHPR deficiency (Crabtree et al., 2009). According to the study results, the diagnosis “HPAВН4C” was confirmed for four patients. The p.Tyr150Cys variant, previously described by Dianzani et al. (1998) in the *QDPR* gene (Dianzani et al., 1998), is classified as a “likely pathogenic” or “pathogenic” variant. In this study and Romstad A.'s paper (Romstad et al., 2000), this variant was found in patients with a pathogenic variant. Also, a premature termination p.Arg221* variant has been previously described by Thöny et al. (1994) and Foroozani et al. (2015).

The p.Leu132= mutation has a high occurrence rate and is registered in several databases, such as dbSNP (rs2597775), ClinVar, Ensembl, PNDdb, Iranom, and others. According to statistics from the Iranom database, the mutation rate of this particular mutation is approximately 0.418. This mutation has been observed in all Iranian ethnic groups, including Arab, Turkish, Azeri, Persian, Baloch, Lur, and Turkmen. Although synonymous variants do not typically result in a change in the amino acid sequence of the protein, they can still impact protein expression, splicing, or folding, which can lead to disease. In the case of the p.Leu132= variant, it is possible that it affects regulatory regions of the gene, which can impact gene expression and lead to disease. Additionally, some synonymous variants can affect RNA splicing, leading to abnormal transcripts and protein isoforms. Therefore, it is possible that the p.Leu132= variant may be disease‐causing through one of these mechanisms. However, further investigation and confirmation through functional studies and clinical observations would be necessary to determine its exact role in disease.

The novel c.645G>T (tgG>tgT) cDNA.681G>Tg.25014G>T gene variant, located in chromosomal location 4:17487221C>A, is also confirmed in the *QDPR* gene by the parents' tests. This variant leads to the replacement of tryptophan (a non‐polar amino acid) with cysteine (a polar amino acid) at position 215 of the DHPR protein. According to the UniProt database, DHPR is a homodimer protein in that each monomer has 8 beta strands and 9 helixes, and Trp215Cys is located in the 9th helix. As a result, the replacement of a polar uncharged amino acid in the site of a non‐polar amino acid changes the correct folding of the protein and likely affects its function. The variant is considered pathogenic in the ACMG classification (PVS1, PM2, and PP1).

The PNDdb database records about 137 mutations for the *QDPR* gene, and most of them are located in the exonic region. However, most of the mutations recorded for this gene in the Iranome database are mutations in the intronic and regulatory regions. In a more comprehensive study published by Hamzehlouei et al. in 2023, 29 variants were recorded for this gene in Iran, 17 of which are new mutations (Ghanei et al., 2023).

According to the results of this study, the incidence of HPABH4A was 7.1%, HPABH4C was 28.4%, and HPAВН4D was 7.1%, while other HPABH4 forms were not detected.

PCD protein has two functions: one, it acts as a dehydratase, and the other, it plays an important role as a binding and dimerization cofactor of HNF‐1α in the nucleus to increase transcriptional activity. According to the UniProt database, this protein has two binding sites for substrate, one at position 61–63 and the other at position 78–81. The PCD protein has 4 exons and includes 315 bases, with exon 1 containing only the ATG start codon. So far, 32 variants have been presented in this gene (Himmelreich et al., 2021). In this study, we also report on an Iranian patient affected by HPA due to a novel mutation in the *PCBD1* gene. A novel frameshift‐causing deletion, c.119delT, was determined in the 40th codon. Mutations in the 40th amino acid can lead to a shorter polypeptide than the native protein. This mutation results in the binding sites of the protein substrate being lost and having no function, which would preclude the regeneration of BH4. Structural and functional studies are required to characterize the pathogenic role of this novel mutation. Most of the reported mutations in the *PCBD* gene are single substitutions or premature stop codons causing benign or transient forms of BH4 deficiency.

## 5 CONCLUSION

This study revealed disease‐causing variants and how they are spread in the *QDPR*, *PTS*, and *PCBD1* genes. These genes are involved in the biosynthesis and recycling of BH4 and are thus necessary for the proper functioning of the PAH enzyme. According to a systematic review study conducted in Iran, the prevalence of *PAH* deficiency was estimated at 93.6% (1133 out of 1210 cases), while the prevalence rates of *QDPR*, *PTS*, and *PCBD1* genes were estimated at 4.1% (50 out of 1210 cases), 2.1% (26 out of 1210 cases), and 0.08% (1 out of 1210 cases) respectively. The study findings also indicated that the percentage of non‐PKU HPA may exceed 2% (Ghanei et al., 2023), which increases the need to pay more attention to these patients and identify the disease‐causing variants. The recently implemented newborn screening programs (NSPs) for PKU in various countries like Iran must include the differential diagnosis of BH4 disorders, establish prompt diagnosis and specific treatment, and prevent neurological sequelae. In this study, the novel c.645G>T, c.373G>A, and c.119delT variants in the *QDPR*, *PTS*, and *PCBD1* genes were present in 3 patients, potentially impacting the function and stability of the BH4. Future in vitro assays may uncover the underlying molecular and functional mechanisms. Raising our awareness of how variants are distributed in the population can open the way for faster diagnosis and the development of new treatments for this disease.

## AUTHOR CONTRIBUTIONS

Mohammad Hamid: conceived the idea, drafted the proposal, and edited the manuscript. Pegah Namdar Aligoodarzi: did the research, analyzed the data, checked the data analysis, and drafted the manuscript. Seyed Reza Kazemi Nezhad: edited the manuscript. Golale Rostami: did the research and analyzed the data. Gholamreza Shariati, Hamid Galehdari, Alihossein Saberi, and Alireza Sedaghat provided the samples and clinical data. All of the authors reviewed and gave the final approval for the paper.

## FUNDING INFORMATION

This work was supported by the Pasteur Institute of Iran [grant number 815].

## CONFLICT OF INTEREST STATEMENT

The authors declare that they have no conflicts of interest.

## ETHICS STATEMENT

This study was approved by the Ethical Committee of the Pasteur Institute, University of Iran.

## Data Availability

The data that support the findings of this study are available from the corresponding author upon reasonable request.
